# Long-Term Outcomes of Anterior Cruciate Ligament Reconstruction Based on Gait Analysis

**DOI:** 10.3390/diagnostics14171977

**Published:** 2024-09-06

**Authors:** Dmitry Skvortsov, Alyona Altukhova, Sergey Kaurkin, Alexander Akhpashev

**Affiliations:** 1 Research and Clinical Centre, 107031 Moscow, Russia; 2 Center for Brain and Neurotechnology, 117513 Moscow, Russia

**Keywords:** knee joint, anterior cruciate ligament, outcomes, gait analysis

## Abstract

Background: Currently available studies on the long-term functional outcomes of anterior cruciate ligament (ACL) reconstruction have yielded conflicting results. The purpose of this study was to evaluate the biomechanical characteristics of walking in the long term after ACL reconstruction. Methods: The study included a test group of 18 patients (3.4 years from the date of ACL reconstruction on average) and a control group of 20 healthy subjects. Their gaits were assessed using functional tests at voluntary walking and fast-walking speeds. The biomechanical assessments utilized included spatiotemporal and kinematic parameters of walking, as well as surface electromyography (EMG) amplitudes of the main flexor-extensor muscles of the lower extremities. Results: Fast-walking speeds and the clearances of the operated-upon limbs in the patient group exceeded those in the control group. The gait cycle in the patient group was significantly longer when walking at a voluntary speed compared to the control group. In the patient group, most of the movements were symmetrical at both speeds, and the parameters did not differ from the control group. The only exception was the hip joint amplitude and the main amplitude of the knee joint flexion, which significantly and simultaneously increased when walking at a fast speed. Conclusions: In the postoperative period, at voluntary speeds, the patient group was characterized by increased amplitudes in the hip and knee joints and higher EMG amplitudes, which almost disappeared at higher speeds.

## 1. Introduction

The number of knee injuries involving damage to the anterior cruciate ligament (ACL) has been increasing in recent times, and the ACL accounts for approximately 50% of all knee injuries (KJ). Most ACL injuries occur among athletes. Female athletes are more susceptible to this injury than male ones [1,2,3]. The risk of this injury in females is estimated to be 3.96 times higher than in males [4]. This is primarily due to the fact that females are biologically more susceptible to sports-related injuries [5].

Several studies have evaluated the effect of ACL ruptures on gait biomechanics. Many authors described different patterns of change in the gait function over time after surgical intervention [6,7,8]. Goetschius J. et al. [9] found decreased amplitudes of flexion, extension, and adduction in the knee joints of injured extremities 1.5 years after ACL reconstruction. Majewska J. et al. [10] examined kinematic changes after ACL reconstruction. They revealed positive functional dynamics in the post-surgery period as represented by a statistically significant increase in the range of motion in the hip and knee joints of both limbs [10]. Erhart-Hledik JC et al. [11] also demonstrated an increase in the flexion angle in the knee joints of operated limbs.

Furthermore, there are studies noting no positive functional dynamics [12]. Milandri G. et al. [13] examined patients post-ACL reconstruction over a long-term period of about 5 years. Shi H. et al. [14] identified a relationship between quadriceps strength asymmetry and knee joint biomechanics asymmetry while walking in subjects who had received ACL reconstruction. Additionally, the function of the hamstring muscles also changed [15]. This muscle group showed excessive and longer-lasting activity that started with a slight delay compared to the healthy control group.

In addition to analyzing walking per se, some researchers used special functional tests to detect minimal borderline changes in gait function. In particular, functional tests with increasing walking speed [16] and lateral single-leg drop landing tests [17] were used to detect near-normal conditions. Increased functional requirements for the operated-upon knee joint during a functional test may allow for the detection of hidden and subtle changes in joint function, which can be useful in studying the long-term outcomes of ACL reconstruction.

Despite the available research on the effect of ACL ruptures on gait parameters, the dynamics of developing changes remain understudied, and the obtained data are contradictory. This lack of knowledge limits rehabilitation and impedes long-term prognoses in patients.

The purpose of this study was to evaluate the biomechanical parameters of gait in terms of long-term outcomes in patients with ACL ruptures.

## 2. Materials and Methods

### 2.1. Patients

The patient group included 18 subjects (13 males and 5 females) with an MRI-confirmed diagnosis of a complete ACL rupture and a subsequent arthroscopic ACL reconstruction surgery. The mean age of the patients was 34.5 ± 10.6 years (range: 17 to 54). The mean period of time after receiving ACL reconstruction surgery was 3.4 years (range: 1 to 10). None of the patients had complaints regarding the knee joint that had received the operation.

The control group included 20 healthy subjects (10 males and 10 females) with a mean age of 28.8 ± 3.6 years (range: 23 to 35) and no history of injuries or diseases of the musculoskeletal system.

This study was conducted based on the ethical principles outlined in the Declaration of Helsinki. Prior to participation in this study, all subjects signed written informed consent forms approved by the Independent Interdisciplinary Committee for Ethical Review of Clinical Research (protocol as of 26 January 2021; State assignment as of 1 January 2021; subject code “Biomechanics-instability”).

### 2.2. Gait Analysis

An impartial assessment of walking was performed using the Stedis complex (Neurosoft, Ivanovo, Russia). With the help of elastic retaining bands, seven inertial sensors were placed on each subject’s sacrum, both outer thighs, the outside of the lateral malleolus of each ankle, and the instep of both feet. Each sensor captured the amplitude parameters of the studied joints and the functional EMG. Disposable surface Mederen electrodes (Tel Aviv-Jaffa, Israel) were used according to SENIAM guidelines [18]. We analyzed the maximum amplitude of each muscle (μV), based on smoothed and rectified EMG values that were normalized to gait cycle, as were the goniograms.

**Figure 1:**
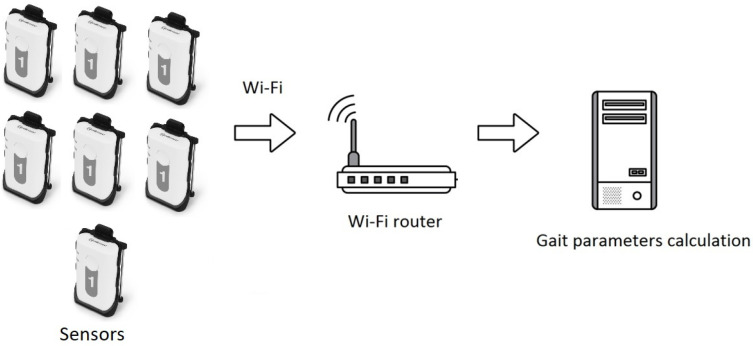
The pipeline diagram showing the process from sensor data acquisition to computer data processing.

Each of the seven inertial sensors had a wireless connection with a computer via a Wi-Fi interface (Figure 1). The accuracy of the synchronization of the data flow between sensors was at least 0.005 s, with the recording frequency of electromyograms being 2000 Hz, and that of the kinematics data, 200 Hz. The neural network for determining gait cycles was trained to detect gait cycles using navigation data [19]. The neural network did not use the EMG signal.

After attaching the sensors and electrodes, the neutral positions of the joints of the lower extremities were recorded with the subject standing upright. The study of biomechanical walking parameters was carried out with the subject walking a distance of 10 m at a voluntary speed. At the end of the distance, the subject made a 180-degree turn in an arbitrary direction and continued moving. The neural network operating in real-time excluded all unsteady or significantly different steps from further analysis. The examination ended when 40 gait cycles had been recorded for each lower extremity.

During the study, the neural network of the software captured the gait cycles (GC) for each lower limb and, in accordance with them, calculated other GC parameters [19].

The final data were generated automatically. This included the values of all recorded parameters of the gait cycle and the automatic determination of the main amplitudes in joint goniograms and envelope EMGs. The EMG data were high-pass filtered at 30 Hz, rectified, and low-pass filtered at 6 Hz to create linear envelopes. All linear envelopes were averaged with respect to the gait cycles. The results of each test were converted into a spreadsheet. The goniograms and EMG linear envelopes of each joint included 200 points per gait cycle.

### 2.3. Study Design

The functional test (FT) with increasing walking speeds [16] was performed at voluntary (self-selected) and fast-walking speeds. The subjects chose both speeds independently.

The following temporal parameters were determined: gait cycle duration (GC, in s), stance phase (SP, % of GC), single support phase (SSP, % of GC), and double support phase (DSP, % of GC).

The following spatial characteristics were determined: clearance (Cl, cm), walking speed (V, km/h), and the rhythm index (RI), which was calculated as the ratio of the larger SP value to the smaller one, in absolute units (seconds).

The following ranges of motion (in degrees) in each joint were recorded: the maximum range of motion for the hip joint (HJ); the amplitudes of first (KJ1) and second (KJ2) flexion in the knee joint; and the maximum amplitude in the ankle joint (AJ).

The tibialis anterior (TA), gastrocnemius (GM), quadriceps femoris (QF), and hamstring (HM) muscles were evaluated for their maximum EMG amplitude developed per gait cycle, measured in μV.

### 2.4. Statistical Analyses

The obtained data were processed by standard methods of ANOVA using the Statistica 12 software package Medians and quartiles (the 25th and 75th percentiles) were calculated. The significance of the differences was assessed using the Mann–Whitney U test with a *p*-value of less than 0.05. Comparisons were performed between affected and unaffected limbs, as well as between patient and control groups.

## 3. Results

The study results are presented in Table 1, Table 2, Table 3 and Table 4. Table 1 shows the means of spatial gait parameters measured at voluntary and fast-walking speeds.

The fast-walking speeds in the patient group significantly exceeded those of the control group (*p* < 0.05). In addition, clearance on the operated side was significantly higher in the patient group than in the control group (*p* < 0.05).

The average values were the same for both limbs, but the level of reliability was achieved only on the operated ones.

At voluntary speeds, the gait cycle in the patient group was significantly longer than in the control (*p* < 0.05). At this speed, the SP and DSP were significantly longer, while the SSP was shorter than that of the control (*p* < 0.05).

In the control group, the increase in walking speed led to a significant decrease in all parameters (*p* < 0.05) except for SSP, which, on the contrary, increased (*p* < 0.05).

No significant differences in the assessed temporal gait parameters were found between the patient and control groups at fast-walking speed (*p* > 0.05).

Table 3 shows the means of assessed movement amplitudes in the lower limb joints.

In the control group, the increase in walking speeds was followed by a significant increase in the hip joint’s range of motion and the knee joint’s main amplitude of flexion during the swing period (*p* < 0.05). The ankle joint’s amplitudes did not change (*p* > 0.05). 

In the patient group, their movements remained unchanged when walking at both speeds, and most values for the affected and unaffected limbs did not differ from the control (*p* > 0.05). The only exceptions were the hip joint amplitudes and the main amplitudes of the knee joint flexion, which significantly and symmetrically increased when walking at a fast speed (*p* < 0.05).

There were no differences between the affected and unaffected limbs in any parameter when walking at a voluntary speed (*p* > 0.05).

The results of the study of EMG muscle amplitudes are presented in Table 4.

In the control group, the increase in walking speed was followed by a significant decrease in the EMG activity of all studied muscles except for the TA (*p* < 0.05). On the contrary, in the patient group, we observed an increase in the EMG activities of all studied muscles. However, in the patient group, the muscle activities of both lower limbs were symmetrical (*p* < 0.05), the same as in the control.

## 4. Discussion

The purpose of this study was to investigate long-term gait outcomes in patients after ACL reconstruction. The higher clearance of the affected limbs when walking at a fast speed is probably non-accidental, which could be indirectly confirmed by the fact that the patient group showed significantly higher amplitudes in the hip joint. In both groups (patient and control), neither the RI nor the clearance changed with an increased walking speed. Thus, the patient group did not differ from the healthy control group in terms of these parameters.

At fast-walking speeds, the temporal parameters in the control group showed decreases in the GC, SP, and DSP muscles, but an increase in the SSP. This is normal for faster walking speeds in healthy individuals [16]. The patients showed similar changes at fast-walking speeds compared to when walking at a voluntary speed. In the patient group, however, the GC duration while walking at a voluntary speed was significantly longer than that of the control group. For all other parameters, the differences between walking at voluntary and fast-walking speeds were similar to those in the control group.

At a fast-walking speed, the values of the parameters in the patient group were similar to those in the control. At a voluntary speed, however, there were differences from the control. Although the voluntary walking speed did not differ significantly between the groups, the GC in the patient group was slightly, though significantly, longer than in the control group, yet it still remained within a normal range. All changes in other GC-related parameters were typical for slower walking, which was actually not the case. An increase in GC duration is usually the result of a decrease in walking speed. If the walking speed remains the same, there can be only one mechanism for increasing the GC time: an increase in the hip joint amplitude. This is exactly what we have noted for walking at both voluntary and fast-walking speeds. At fast-walking speeds, however, the temporal parameters of walking did not differ between patients and controls. Another study [20] also used a walking test at different speeds. However, data comparisons between that study and ours may be invalid because of significant differences between the patient populations: 29% of patients in that study [20] experienced discomfort when walking fast. Additionally, that study used a treadmill test, which itself causes certain changes in walking parameters.

In the control group, the analysis of movement amplitudes in the joints showed that an increase in the walking speed was associated with an increase of the amplitudes in the hip joints and the swing amplitudes in the knee joints. Walking at both speeds showed no asymmetry in any of the analyzed parameters.

In the patient group, the following results were obtained: at voluntary walking speeds, the amplitudes in the hip joints and the swing amplitudes in the knee joints were significantly higher than in the control group. Another study [21] found no decrease in the range of motion in the hip and knee joints after surgery. The study population consisted of female athletes. The authors noted a trend of decreased amplitudes in hip joints, which did not reach significant values. In our study, we observed an increase in the amplitudes of hip joints while walking at voluntary speeds. At fast-walking speeds, the same parameters were again similarly and significantly higher than in the control group. Thus, in the patient group, a slightly modified hip and knee joint function pattern can be noted, characterized by an increase in the range of motion when walking at both speeds. Our results on the kinematics of flexion-extension movements in the knee joint and the duration of the GC are consistent with those of another study [22].

In the control group, during fast-walking, the EMG activities of all analyzed muscles decreased, with the only exception being the TA, whose amplitude increased. In the patient group, higher walking speeds increased the activities of all these muscles. However, there was full symmetry between the affected and unaffected limbs. In their analytical review Sherman DA et al. [15],. noted excessive and longer activity in hamstring muscles that occurred with a slight delay compared to healthy individuals. Such an effect increases the risk of subsequent knee injuries.

The results of the functional tests were consistent with the study by Stoelben KJV et al. [22], who noted that 4 years after ACL reconstruction, the walking kinetics of patients largely returned to normal. Therefore, at higher speeds, higher moments of force were observed, which is also typical for healthy individuals. Thus, our study has demonstrated that the outcome of ACL reconstruction can be a full recovery of both walking function and the operated-upon joint itself. This is consistent with other authors [10,11] and with our previous research [23]. Our findings do not support the conclusions [24,25,26] that ACL repair does not restore knee joint biomechanics. The current study has found a certain modification in walking biomechanics that is characterized by symmetry. Other research methods allowed the detection of other functional nuances. For example, Webster KE et al. [27] discovered an asymmetry of the moment of forces in the hip joint at the beginning of the support phase. Two years after ACL reconstruction, the vertical support reaction was greater in the operated extremity [27,28,29]. Noehren B et al. [21] achieved similar results with the same method.

The need to conduct special functional tests in patients of this category is also beyond doubt. Where the pathology is not obvious, functional tests with changes in walking speed [30] or with uphill and downhill walking [20] make it possible to identify existing changes at an early stage.

This study has a number of limitations. First of all, the number of included patients was rather small because it is quite difficult to enroll patients with no obvious consequences of past trauma. For this reason, there is a fairly large scatter in both the time elapsed after reconstruction and in the age of those examined.

The long-term outcomes of ACL reconstruction are characterized by fully symmetrical and normal walking function of the lower limbs. Thus, the functions of the operated-upon and non-operated-upon limbs are indistinguishable. However, walking function is slightly modified in the long term after ACL reconstruction. Patients with a history of ACL surgery showed higher maximum amplitudes in the hip and knee joints, as well as higher amplitudes in the tibialis anterior, gastrocnemius, and hamstring muscles.

## 5. Conclusions

The current study found a restructuring of gait biomechanics occurring in the patient population while walking at voluntary speeds. Being close to normal limits, it was characterized by increased amplitudes in the hip and knee joints, which made it possible to maintain the same speed with slight increase in the durations of GC, SP, and DSP. This is a simple and natural mechanism to reduce the load on the operated limb. Increases in amplitudes in the hip and knee joints were typical for fast-walking speeds as well.

Based on the bioelectrical activities of muscles, a fast-walking speed in the control group led to a decrease in the activities of GM, QF, and HM. In the patient group, the activities of all muscles increased during fast-walking.

Thus, even in the long-term period after ACL reconstruction, there still was a fairly delicate and simultaneous modification of parameters when walking at a voluntary speed, which almost disappeared at higher speeds.

## Figures and Tables

**Table 1:** Spatial gait parameters at voluntary and fast-walking speeds.

Parameter	V km/h	RI	Cl (cm)	
			Affected	Unaffected
Voluntary, patients	4.3 [4.0; 4.8]	0.98 [0.96; 0.98]	15.0 [14; 16]	15.0 [13; 16]
Voluntary, controls	4.3 [4.1; 4.7] *	0.98 [0.97; 0.99]	14 [12; 15]	
Fast-walking, patients	6.1 [5.8; 6.5] ^#^	0.97 [0.96; 0.98]	14.5 [13; 15] ^#^	14.0 [12; 15]
Fast-walking, controls	5.6 [4.4; 6.1]	0.98 [0.97; 0.99]	13 [11; 14]	

*—significant difference from fast-walking speed in control (*p* < 0.05); ^#^—significant difference from voluntary speed in control (*p* < 0.05).

**Table 2:** Temporal gait cycle parameters at voluntary and fast-walking speeds.

Parameter	GC (c)		SP (%)		SSP (%)		DSP (%)	
	Affected	Unaffected	Affected	Unaffected	Affected	Unaffected	Affected	Unaffected
Voluntary, patients	1.2 [1.1; 1.2] *^$^	1.2 [1.1; 1.2] *^$^	63.2 [62.6; 65.4] *	64.4 [63.4; 64.7] *^$^	35.7 [34.9; 36.7] *^$^	36.4 [34.6; 37.3] *^$^	27.6 [25.9; 30.7] *^$^	27.6 [25.7; 30.6] *^$^
Voluntary, controls	1.1 [1.0; 1.1] ^#^		62.9 [61.6; 63.7] ^#^		37.4 [36.3; 38.3] ^#^		25.4 [24; 27.7] ^#^	
Fast-walking, patients	1.0 [0.9; 1.0]	1.0 [0.9; 1.0]	61.0 [59.9; 62.2]	61.6 [60.8; 62.7]	38.3 [37.3; 38.9]	39.1 [37.8; 39.7]	22.6 [21.2; 24.1]	22.8 [21.5; 24]
Fast-walking, controls	1.0 [0.9; 1.0]		61.4 [60.3; 62.9]		38.4 [36.8; 39.2]		23.1 [21.7; 26.3]	

*—significant difference from fast-walking patients (*p* < 0.05); ^$^—significant difference from controls walking at voluntary speed (*p* < 0.05); ^#^—significant difference from fast-walking controls (*p* < 0.05).

**Table 3:** Amplitudes in lower limb joints at voluntary and fast-walking speeds (in degrees).

Parameter	HJ		KJ1		KJ2		AJ	
	Affected	Unaffected	Affected	Unaffected	Affected	Unaffected	Affected	Unaffected
Voluntary, patients	36.5 [35.0; 41.0] *^$^	38.0 [36.0; 41.0] *^$^	15.5 [12.4; 17.9]	15.4 [11.6; 18.0]	60.5 [57.9; 66.9] ^$^	61.5 [56.0; 66.1] ^$^	29.0 [25.0; 37.0]	30.5 [27.0; 35.0]
Voluntary, controls	32.4 [30.6; 36.8] ^#^		14.4 [12.3; 17]		51.2 [46.0; 56.7] ^#^		32.8 [30.5–36.9]	
Fast-walking, patients	43.5 [42.0; 47.0] ^%^	44.0 [41.0; 49.0] ^%^	15.1 [13.0; 17.3]	16.4 [13.0; 18.5]	58.4 [56.8; 59.9] ^%^	61.5 [54.8; 62.5] ^%^	31.5 [27.0; 37.0]	31.0 [27.0; 36.0]
Fast-walking, controls	40.0 [34.5; 44]		14.4 [11.7; 17.5]		54.8 [49.7; 60.5]		33.0 [29.0; 37.0]	

*—significant difference from fast-walking patients (*p* < 0.05); ^$^—significant difference from controls walking at voluntary speed (*p* < 0.05); ^#^—significant difference from fast-walking controls (*p* < 0.05); ^%^—significant difference from fast-walking patients vs. controls (*p* < 0.05).

**Table 4:** EMG amplitudes of walking at voluntary and fast-walking speeds (in µV).

Parameter	TA		GM		QF		HM	
	Affected	Unaffected	Affected	Unaffected	Affected	Unaffected	Affected	Unaffected
Voluntary, patients	201.0 [150.0; 249.0] *	230.0 [116.0; 280] *	164.0 [121.0; 202.0] *	169.0 [134.0; 219.0] *	60.0 [47.0; 73.0] *	65.0 [52.0; 93.0] *	113.0 [87.0; 159.0] *	111.0 [80; 153.0] *
Voluntary, controls	251.0 [179.5; 313.5] ^#^		226.5 [171.0; 298.0] ^#^		114.0 [81.0; 154.5] ^#^		125.5 [99.5; 162.0] ^#^	
Fast-walking, patients	332.0 [210.0; 413.0]	353.0 [182.0; 435.0]	274.0 [168.0; 471.0]	251.0 [204.0; 464.0]	112.0 [98.0; 164.0]	132.0 [93.0; 216.0]	186.0 [136.0; 367.0]	186.0 [105.0; 247]
Fast-walking, control	131.5 [109.5; 161.0]		114.0 [85.0; 140.0]		56.0 [28.5; 78.5]		74.0 [48.0; 98.5]	

*—significant difference from fast-walking in patient group (*p* < 0.05); ^#^—significant difference from controls at fast-walking speed (*p* < 0.05).

## Data Availability

All data are available at https://doi.org/10.17632/g95wxtbkb4.1.
